# Bridging experimental biology and clinical practice: lessons from autoinfective larvae of *Strongyloides stercoralis*

**DOI:** 10.1128/jcm.01411-25

**Published:** 2025-11-13

**Authors:** Vitor Luís Tenório Mati

**Affiliations:** 1 Departamento de Medicina, Universidade Federal de Lavras 67739, Lavras, Minas Gerais, Brazil; Mayo Clinic Minnesota, Rochester, Minnesota, USA

**Keywords:** life cycle, diagnostic paradigms, pulmonary development, autoinfective larvae, strongyloidiasis

## LETTER

Since the pioneering investigations of the life cycle and intricate pathophysiology of *Strongyloides stercoralis* (1, 2), researchers, clinicians, and laboratory diagnosticians have continually grappled with the challenge of fully understanding this parasite. In a recent step forward, Zhao et al (3) used classical morphological analysis together with modern techniques to redescribe the autoinfective fourth-stage larvae (L4) isolated from respiratory specimens of infected individuals. Their study tacitly revitalizes the academic discussion around the developmental stages and migratory routes of *S. stercoralis*, which are rendered complex by the nematode’s singular autoinfective capacity—a phenomenon associated with hyperinfection and dissemination syndromes (4–6). The authors reference 2 antecedent experimental studies with evidence of intermediates between the infective third-stage larva (L3) and the adult female in host pulmonary tissues—1 conducted in dogs (2) and another in marmosets, a nonhuman primate model (7)—thus illuminating substantive lacunae in current knowledge, although their discussion falls short of a rigorous and integrative interpretation of these findings.

Faust (2), for example, delineates the ontogenetic sequence in canine hosts as follows: “infective filariform larvae” migrate from the integument to the pulmonary parenchyma, where they transition into “post-filariform larvae”—forms still bearing notched tails and indeterminate sexual phenotype, primarily resident in lung tissue—followed by “pre-adolescent females” inhabiting both lungs and gastrointestinal tract, and culminating in “mature females” in the intestines, with occasional presence in the lungs and diverse viscera. These “post-filariform larvae”—and especially the “pre-adolescent females”—closely mirror the morphological hallmarks of the L4 stage as redescribed, exhibiting an esophagus extending half the body length, a prominent anal opening, and inception of the genital primordium, albeit with lingering debate as to the precise definition of caudal morphology (2, 3). Contrariwise, the morphometrics of the early “post-filariform larvae” are more consonant with the L3 profile (2, 6, 8). In nonhuman primate hosts undergoing disseminated infection, multiple larval morphotypes (eg, rhabditiforms, autoinfective filariform L3 and L4, and juvenile females equivalent to the “adolescent female” described by Faust, with advanced ovarian differentiation, a prominent vulva, and a smooth caudal contour) have been documented in various organs, including lungs and trachea (7). These observations underscore the imperative for deeper inquiry into the morphological and biological nuances—especially the developmental idiosyncrasies—of larval and juvenile forms in human strongyloidiasis, a prerequisite for advancing precise diagnostic criteria and robust clinical management, as well as a more sophisticated comprehension of the nematode’s ontogeny.

Within this framework, the debate regarding the lungs as a site of larval maturation and the putative necessity of pulmonary passage within the life cycle of *S. stercoralis* is reignited. Conventionally, infective L3 is understood to traverse the bloodstream to the lungs, ascend the airways, and ultimately mature within the intestine, albeit with occasional deviations into extra-pulmonary niches (4–6). Still, the anatomical locus of the L3 to L4 molt is contentious: while rodent data (eg, *Strongyloides ratti*) suggest that molting transpires exclusively in the intestine (9), interspecies peculiarities preclude direct extrapolation to *S. stercoralis*. Already in the early 20th century, Fülleborn (1914) (1) and Faust (1933) (2) identified adult nematode females within the canine pulmonary tree—a finding that has since been experimentally corroborated—intimating that development and ecdysis may indeed occur in lung parenchyma, contingent on host species and pathophysiological context, especially in settings of immune dysregulation, which can destabilize host-parasite equilibrium and foster aberrant migratory pathways (4, 6, 7).

Experimental paradigms reflect findings in human pathology, though evidence remains somewhat fragmented in the literature. Clinical data from sputum analysis—an underexploited yet informative diagnostic resource—validate the conjecture of pulmonary maturation. Reports of L3 in pulmonary samples are not infrequent (5, 10–12), corroborating our regional observations (Minas Gerais, Brazil), but the detection of rhabditiform larvae (5, 13, 14), as well as eggs or early larvae in sputum cultures (15), collectively strengthen the hypothesis of *in situ* pulmonary development. The seminal documentation by De Paola (16) of parthenogenetic female in lung tissue at autopsy—later substantiated by subsequent severe strongyloidiasis cases (17–19)—extends these pathobiological inferences. Despite some reservations expressed (20), the cumulative weight of these descriptions—including L4 retrieval from sputum (3)—concretizes the intermediary role of pulmonary tissues in the life cycle and pathology of *S. stercoralis*. Thus, pulmonary larval ecdysis is demonstrably plausible, and there is compelling evidence for the local maturation of parthenogenetic female within the lung. Nevertheless, passage through the lung remains a facultative, not an invariant, requirement for parasite development, with the timing of molts being influenced by intrinsic parasite biology, infection dynamics, and especially host immune competence (4, 7). Awareness of such patterns is critical in the setting of severe or disseminated disease. The presence of autoinfective filariform larvae (L3 and/or L4)—particularly in abundance and in patients without recent epidemiological exposure—serves as a sensitive marker of immune perturbation and should trigger a directed evaluation for immunosuppression (such as corticosteroid use, malignancies, or HTLV-1 infection) (5, 6).

While the advent of molecular methods holds considerable promise, classical parasitological techniques remain indispensable pillars of diagnosis (3). Standard approaches rely on the detection of larvae in fecal samples and, when clinically indicated, in bronchoalveolar lavage fluid or sputum. It is crucial to invest in advanced training for the precise identification of larval stages and for the nuanced interpretation of these findings—always contextualized within the clinical and immunological landscape. In summary, the developmental biology and migratory dynamics of *S. stercoralis*—encompassing both canonical and atypical routes—reveal a complexity that far surpasses common perception. Addressing the need for refined diagnostic paradigms that harmonize classical expertise with technological advancements is essential for implementing standardized protocols and achieving effective clinical management. The ongoing preservation, cultivation, and dissemination of parasitological knowledge—coupled with the seamless integration of foundational research into clinical practice—remain vital for accurate diagnosis and, ultimately, improved global health outcomes for those affected by human strongyloidiasis.
